# *MET* exon 14 mutations as targets in routine molecular analysis of primary sarcomatoid carcinoma of the lung

**DOI:** 10.18632/oncotarget.16403

**Published:** 2017-03-21

**Authors:** Raphaël Saffroy, Vincent Fallet, Nicolas Girard, Julien Mazieres, Denis Moro Sibilot, Sylvie Lantuejoul, Isabelle Rouquette, Françoise Thivolet-Bejui, Thibaut Vieira, Martine Antoine, Jacques Cadranel, Antoinette Lemoine, Marie Wislez

**Affiliations:** ^1^ Department of Biochemistry and Oncogenetics, AP-HP Hôpital Paul-Brousse, Hôpitaux Universitaire Paris Sud, Villejuif, France; ^2^ UMR-S 1193, Univ Paris-Sud, Université Paris-Saclay, Villejuif, France; ^3^ GRC n°04, Theranoscan, Sorbonne Universités, UPMC Univ Paris 06, Paris, France; ^4^ Pulmonary Medicine Unit, AP-HP, GH HUEP, Hôpital Tenon, Paris, France; ^5^ Respiratory Medicine, Thoracic Oncology Group, Groupement Hospitalier Est, Hospices Civils de Lyon, Lyon, France; ^6^ Pulmonary medicine Unit, Hôpital Larrey, Centre Hospitalier Universitaire, Toulouse, France; ^7^ Thoracic Oncology Unit, PTV, CHU Grenoble Alpes, Grenoble, France; ^8^ Department of Pathology, Hôpital A. Michallon, Centre Hospitalier Universitaire, Grenoble, France; ^9^ Centre Hospitalier Universitaire Rangueil, Service d’anatomie Pathologique, Toulouse, France; ^10^ Department of Pathology, Hôpital Louis Pradel, Lyon, France; ^11^ Department of Pathology, AP-HP, GH HUEP, Hôpital Tenon, Paris, France

**Keywords:** sarcomatoid carcinomas of the lung, non-small cell lung cancer, MET exon 14 skipping mutation

## Abstract

MET exon 14 splicing mutations are new targetable oncogenic drivers reported in 3% of non-small cell lung cancer (NSCLC) cases and have been shown to be more common in pulmonary sarcomatoid carcinomas (PSCs). This study sought to screen mutations affecting MET exon 14 splice sites in a large SC cohort of Caucasian patients, with a large adenocarcinoma cohort as internal control.

We tested 81 patients with SC and 150 with adenocarcinoma for splice site DNA mutations leading to RNA splicing-based skipping of MET exon 14. To this end, we employed a mass spectrometry-based custom-designed PCR assay for routine analysis of whole MET exon 14 and flanking intronic regions using formalin-fixed paraffin-embedded (FFPE) tumor samples.

Our results revealed a 4.9% mutation rate for MET exon 14 mutations in Caucasian SC patients, which is, though highly variable, within the usual range reported in NSCLC. Discrepancies with previous results reported in SC could be accounted for the small number of cases, ethnicity, epithelial component, and percentage of other driver mutations, such as KRAS, in the patient populations studied. Based on our study findings, SC patients should be screened for MET exon 14 mutations in the same manner as adenocarcinoma patients.

## INTRODUCTION

Somatic mutations of the *MET* gene have been described in non-small cell lung cancers (NSCLC) as new promising targets for small-molecule kinase inhibitors and monoclonal antibodies targeting *MET* or its ligand. Amongst mutations associated with oncogenic activation that are actionable by targeted therapies, those affecting exon 14 splice sites have been recently described [1, 2]. Patients may thus be screened on a routine basis and then be treated with MET inhibitors like crizotinib [3] as appropriate. However, the diverse compositions of the MET exon 14 splice sites and their variable locations in intronic regions require the examination of large and new sequences by high-throughput sequencing or genotyping technologies using formalin-fixed paraffin-embedded (FFPE) tumor samples.

Somatic mutations leading to MET exon 14 splicing have been shown to occur in approximately 3% of NSCLC [1] cases, with a higher frequency observed in primary sarcomatoid carcinomas (SC) of the lung [4], although reports were based on highly variable cohort sizes and sequencing technologies (Table 1). Pulmonary SC is a rare tumor, accounting for less than 3% of NSCLC [5] cases. Their poor prognosis and resistance to conventional chemotherapy [6], however, pose a therapeutic challenge. Similarly to adenocarcinomas, the development of molecular biology over the past five years has enabled us to gain knowledge of specific aberrations in SC genomes as new therapeutic targets. Recently, we have shown that SC tumors exhibited high mutation rates [7], which likely increase tumor immunogenicity, rendering them good candidates for immunotherapy.

**Table 1:** Studies assessing MET mutations in sarcomatoid carcinoma

Study	Number of pts *N*=	Histological Subtypes of SC	Controls *N*=	MET ex 14 analysis	MET ex 14 frequency in SC *N* (%)	MET ex 14 frequency in controls *N* (%)
**Saffroy *et al* 2016**	81	PC (77.8%) Others (22.2%)	ADC (N=150)	Whole met ex 14 and flanking intronic regions (14 +/− n bp)MassArray and HRMParaffin embedded tumors	4 (4.9%)	8 (5.3%)
**Schrock *et al* 2016**	104	PC and others	NSCLC (N=11 101) includingADC (N=7140)	NGS - Capture hybrydization including intronic regionsParaffin embedded tumors	8 (7.7%)	NSCLC : 290 (2.14%)ADC : 205 (2.8%)
**Tong *et al* 2016**	22	ND	NSCLC (N=665) including ADC (N=392)	Whole met ex 14 and flanking intronic regions (14 +/− n bp) Sanger sequencingParaffin embedded tumors	7 (31.8 %)	NSCLC : 1 (0.3%)ADC : 10 (2.6%)
**Awad *et al* 2016**	15	ND	NSLC (N=1126) including ADC (N=873)	NGS (22 genes)	4 (26.7%)	NSCLC : 6 (2.4%)ADC : 18 (2.1%)
**Liu *et al*2015**	36	ND carcinosarcoma and blastoma excluded	Not studied	Whole-exome sequencingTargeting exome sequencing (Truseq panel, Illumina) Sanger sequencing half frozen & half paraffin embedded tumors	8 (22%)	Not studied
**Vieira *et a*l 2014**	77	PC (78%) Others (22%)	Not studied	Sizing analysis of fluorescently labeled PCR products (only 3′-splice site of MET ex 14 deletions) Formalin-fixed paraffin-embedded samples	2 (3%)	Not studied

Pts : patients; SC : sarcomatoid carcinomas; ADC : adenocarcinoma, NSCLC : non small lung carcinoma; PC: pleomorphic carcinoma; ND : Not described; CGP : comprehensive genomic profiling; NGS: next-generation sequencing; ex : exon;

Due to a new promising oncogenic driver potentially targeted by MET inhibitors, a molecular diagnosis strategy combining a highly sensitive and specific iPLEX chemistry and mass spectrometry-based panel was designed by our team for the synchronous screening of the actionable MET sequence abnormalities resulting in various exon 14 splicing alterations in FFPE samples of limited tissue. This technique was combined with another complementary one based on the use of HRM (High resolution Melting) detection to screen each DNA sample for the presence of sequence abnormalities that could not have been detected by the spectrometry-based Massarray panel. We have applied these techniques to the screening of a multicenter French cohort with primary pulmonary SC as previously characterized [7] and of a prospective cohort with primary pulmonary adenocarcinomas as internal control.

We tested 81 patients with SC and 150 with adenocarcinoma for somatic mutations affecting both splice sites and intronic non-coding regions immediately adjacent to exon 14 of the *MET* gene. Results were compared to fluorescence *in situ* hybridization (FISH) analyses and clinicopathological characteristics.

## RESULTS

### Patient characteristics

Clinical characteristics of SC patients are presented in Table 2. Median age was 61 years (range 41-79). Patients were more commonly males (74.1%) and heavy smokers (smokers: 92.6%; median pack-year: 36; range: 1-100). They were almost all Caucasian (80.2%) and none was Asian. The majority of patients (61.7%) underwent lobectomy. In terms of pathological stage, 15 patients were Stage I (18.5%), 24 Stage II (29.6%), 35 Stage III (43.2%), and seven Stage IV (8.6%). Surgery performed on Stage IV patients was mainly for diagnostic purposes. Pleomorphic carcinoma was found to be the main histological subtype (77.8%). No patient had been pretreated with tyrosine kinase inhibitors (TKI) or targeted therapy.

**Table 2:** Clinical characteristics of patients with pulmonary sarcomatoid carcinoma (*n=81*)

Variable	Total (%)
**Gender**	
Male	60 (74.1%)
Female	21 (25.9%)
**Age (years)**	
Median (range)	61 (41-79)
Ethnicity*	
Caucasian	65(80.2%)
Other	10 (13.3%)
Asian	0 (0%)
Smoking history**	
Smoker	75 (92.6%)
Non-smoker	5 (6.2%)
**Clinical Stage**	
I- II	39 (48.1%)
III-IV	42 (51.9%)
**Histological subtype**	
Pleomorphic carcinoma	63 (77.8%)
Spindle cell carcinoma	4 (4.9%)
Giant cell carcinoma	6 (7.4%)
Spindle and giant cell carcinoma	3 (3.7%)
Carcinosarcoma	4 (4.9%)
Pneumoblastoma	1 (1.2%)
**Type of surgery**	
Pneumonectomy	17 (21%)
Lobectomy	50 (61.7%)
Other	14 (17.3%)
**Neoadjuvant chemotherapy**	16 (19.8%)

*6 data missing - ** 1 data missing

Clinical characteristics of patients with adenocarcinoma are presented in Table 3. Median age was 62 years (range 28-86). Patients were more commonly males (66%) and smokers (77.3%). They were almost all Caucasian (85.3%) and two were Asian (1.3%). In terms of pathological stage, they were almost all locally advanced or at metastatic stage (94.7%). No patient had been pretreated with TKIs.

**Table 3:** Characteristics of patients with pulmonary adenocarcinoma (*n=150*)

Variable	Total *n* (%)
**Gender**	
Male	99 (66%)
Female	51 (34%)
**Age (years)**	
Median (range)	62 (28-86)
**Ethnicity**	
Caucasian	128 (85.3%)
Other	20 (13.3%)
Asian	2 (1.3%)
**Smoking history**	
Never	34 (22.7%)
Smoker	63 (42%)
Ex-smoker	53 (35.3%)
**Clinical Stage**	
I-II	8 (5.3%)
III-IV and metastatic-relapse	142 (94.7%)
**Sample origin**	
**Primary tumor**	118 (78.7%)
Mediastinal node	15 (10%)
Metastasis	17 (11.3%)
**Routine screening mutations**	
No mutations	100 (66.7%)
EGFR	14 (9.3%)
KRAS	35 (23.3%)
BRAF	1 (0.7%)
HER2	0
PI3KCA	0

The 81 SC tumors were previously characterized for oncogenic driver mutations using the LungCarta Panel [7]. Among them, 56 (69.1%) harbored at least one mutation, the most common being *KRAS* (27.2%). *EGFR* mutations were present in 22.2%, *PI3KCA* in 4.9%, and *BRAF* in 2.5%. The *EGFR* mutations were almost always rare mutations (89%). In 32 tumors (39.5%), two or more mutations co-existed [7].

Among the 150 adenocarcinoma tumors routinely screened for actionable oncogenic driver mutations, 50 (33.3%) harbored one mutation. The most common were *KRAS* (23.3%), *EGFR* (9.3%), and *BRAF* (0.3%). No *PI3KCA* mutation was found.

### *MET* mutations

**Figure 1:**
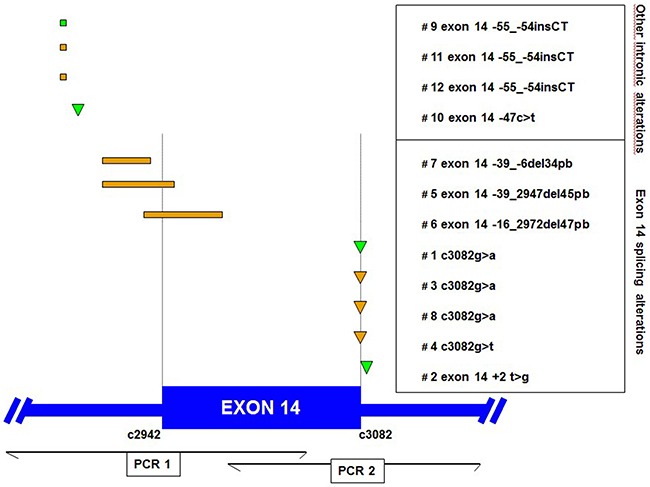
Locations of *MET* exon 14 genomic alterations found in sarcomatoid (green) and adenocarcinoma (orange) patients The positions of each *MET* mutation are displayed in relation to the *MET* gene. Deletions and insertions are shown as rectangles, and point mutations are shown as triangles. The table indicates the patient number and the nucleotide position of each mutation. Position of amplicons for PCR 1 and 2 are shown down.

*MET* gene sequence abnormalities are shown in Figure 1. Nine different gene variants were found. There were four SC (4.9%) and eight adenocarcinomas (5.3%) exhibiting *MET* exon 14 mutations.

Of the 81 pulmonary SC, the four MET sequence abnormalities (4.9%) occurred in patients with a median age of 67 years, of whom three were female, three smokers, and one ex-smoker (Table 4). Somatic *MET* mutations were almost always found in tumors without epithelial components, *i.e*., in three patients with giant cell carcinoma, yet only in one with pleomorphic carcinoma. There were two MET exon 14 splice site mutations (2.5%) and two intronic mutations at 47 and 54kb, respectively. MET exon 14 skipping mutations were not mutually exclusive with *KRAS* and *EGFR* mutations in SC. In one case, exon 14 splicing alteration was associated with KRAS G12V mutation and in another one with a rare EGFR mutation (G719A).

**Table 4:** Individual characteristics of patients with MET exon 14 splice site mutations

Patient	cDNA *MET* position*	Chr7 startposition**	Chr7 endposition**	Age (Years)	Gender	Ethnicity	Smoking history	Histology	Othermutations
#1	c3082g>a;	116771987	116771987	75	Male	Caucasian	Current	SC Spindle and giant cell carcinoma	EGFR G719A
#2	exon 14 +2 t>g	116771989	116771989	65	Female	Caucasian	Current	SC Pleomorphic carcinoma (ADK and Spindle cell carcinoma)	-
#9	exon 14 -55_-54insCT	116771796	116771797	66	Female	Arab	Current	SC Giant cell carcinoma	-
#10	exon 14 -47c>t	116771802	116771802	68	Female	Caucasian	Ex-smoker	SC Giant cell carcinoma	KRAS G12C
#3	c3082g>a;	116771987	116771987	84	Female		Never smoker	ADC	-
#4	c3082g>t;	116771987	116771987	64	Female		Ex-smoker	ADC	-
#5	exon 14 -39_2947del45pb	116771810	116771854	79	Male	African	Never smoker	ADC	-
#6	exon 14 -16_2972del47pb	116771833	116771879	81	Female		Never smoker	ADC	-
#7	exon 14 -39_-6del34pb	116771810	116771843	60	Female		Never smoker	ADC	-
#8	c3082g>a;	116771987	116771987	63	Male		Current	ADC	-
#11	exon 14 -55_-54insCT	116771796	116771797	39	Male		Current	ADC	-
#12	exon 14 -55_-54insCT	116771796	116771797	77	Male	African	Current	ADC	-

SC: sarcomatoid carcinoma; ADC: adenocarcinoma

*cDNA according to COSMIC nomenclature

** gDNA according to www.genome.ucsc.edu (Dec2013) nomenclature

Of the 150 consecutive adenocarcinomas, eight *MET* sequence abnormalities (5.3%) occurred in patients with a median age of 70 years, of whom four were female, three smokers, one ex-smoker, and four never smokers (Table 4). There were six *MET* exon 14 splice site mutations and two intronic mutations at 52 and 54kb, respectively. Interestingly, the *MET* mutations were exclusive with *EGFR, KRAS, BRAF, HER2* or *PI3KCA* mutations. Considering the adenocarcinomas without any actionable EGFR or BRAF mutations, the percentage of tumors exhibiting MET exon 14 splice mutations was eight out of 100 patients.

### *MET* FISH results

*MET* FISH was assessed for 71 cases of the SC cohort (Supplementary Table 1). In total, 14 patients (19.7%) were found to be *MET* FISH-positive (mean ≥5 copies per cell), including six patients (8.4%) with true gene amplification. Among the four patients with *MET* exon 14 mutation, three were *MET* FISH-negative and one *MET* FISH-positive, with a mean copy number of 5.2 and a MET/CEP7 ratio of 1 (polysomy).

## DISCUSSION

We have screened a French cohort involving 81 SC for *MET* exon 14 splice site mutations in order to determine the frequency of patients who would benefit from anti-MET therapies in clinical practice. Our internal control involved 150 patients with adenocarcinomas prospectively screened for actionable mutation using our molecular platform. The frequency of *MET* exon 14 splice site mutations was 4.9% for SC and 5.3% for adenocarcinoma patients.

*MET* oncogenic mutations leading to diverse exon 14 splicing alterations are emerging as a new hope for patient subsets likely to derive benefit from targeted therapies, especially those with SC. The splice site DNA mutations result in RNA splicing-based skipping of *MET* exon 14, corresponding to the receptor's juxta-membrane portion. These somatic mutations have been reported in both primary lung cancer specimens and lung cancer cell lines since 2003[8, 9]. They were reported to increase MET stability by decreasing the protein ubiquitination, thus prolonging receptor signaling upon HGF stimulation. MET inhibitors like crizotinib and cabozantinib showed effectiveness in patients with mutations at the MET exon 14 splice sites [1].

More recently, several large series assessing the genomic landscape of lung adenocarcinoma reported MET exon 14 skipping mutations to occur in approximately 3% of all tumors [1, 2, 10, 11] and 2% of other lung neoplasms [1]. However, in smaller SC series, different prevalence rates have been reported, ranging from 3%[12] to 22%[4]. These discrepancies could be due to the small number of patients studied in some series but also to heterogeneous geographical origin (Asian versus non-Asian, as already shown for EGFR mutations), histological description (presence or absence of epithelial components), or prevalence of other driver mutations like *KRAS* mutations in the series. Furthermore, these series used many different technologies and bioinformatics software for genetic testing or selection of MET gene variants from sequencing reads, respectively, with most of them being poorly described. Of 81 pulmonary SC, the frequency of MET exon 14 splice site mutations was 4.9%, *i.e*., the lowest rate yet reported, although this value is very close to the 7.7% rate recently reported by Schrock *et al*. for an American cohort of lung cancers including 104 SC analysed by a well-validated hybrid capture-based NGS [13].

Concerning ethnicity, almost all of our patients were Caucasian and none Asian, although a higher *MET* exon 14 frequency was reported in one Asian series, with a smaller patient number [4]. In another series, limited clinical and histopathological data [1] was provided [2], without any details on the ethnic origins, thus impeding us to find a correlation between the high MET mutation rate and Asian ethnicity.

The proportion of sarcomatoid histological subtypes, such as pleomorphic carcinomas, or the presence or absence of adenocarcinomas in the epithelial component, might also have impacted the MET exon 14 rate. The recent results of Schrock *et al*., showing similar frequency of MET exon 14 skipping alterations in Caucasian patients with similar histological descriptions, tend to confirm this hypothesis for SC. In our study, the proportion of patients with pleomorphic carcinoma (77%) was lower than that of the cohorts studied by Awad *et al*. (100%) [2] and by Liu *et al*. (93%) [4]. In these two previous cohorts, adenocarcinoma was however the predominant epithelial component (100% and 79%, respectively), contrary to our study with only 47% according to morphology and immunohistochemistry [7].

Lastly, we cannot exclude that the frequency of MET mutations differed depending on other driver mutations. For example, there was a higher rate of *KRAS* mutated sarcomatoid tumors in our series (27%, *n=22*) than in Liu's and Awad's cohorts (15%, *n=6;* 20%, *n=3*, respectively) [2, 4]. The high *KRAS* mutation rate in our SC cohort was similar to that previously described. This may account for the lower *MET* mutation rate observed in our study. This may also suggest that the high *MET* exon 14 mutation rates reported in Liu's and Awad's cohorts could be related to their highly selected study populations.

As exon 14 skipping mutations are sensitive to MET inhibitors, this new oncogene driver should be detected in clinical routine practice. For our routine practice, we have thus used a diagnosis strategy combining two techniques suitable for FFPE tumor biopsy genetic testing with a high sensitivity and specificity, enabling us to detect all gene abnormalities leading to MET exon 14 splicing. As reported by other studies, several different mutation types in MET exon 14 and its flanking introns were detected [2, 4]. Splice site DNA mutations leading to RNA splicing-based skipping of MET exon 14 comprise base substitutions, deletions, insertions, or complex indels that could be randomly located at splice acceptor or donor sites. This may also apply to the app. 25 bp intronic non-coding regions immediately adjacent to the splice acceptor site. Other mutations occurred deeper within intron 13, without overlapping with the splice acceptor site. Therefore, the full screening of MET gene abnormalities proves difficult in routine practice. This must be taken into account when designing clinical diagnostic sequencing assays aimed to capture or genotype all possible activating MET mutations. In our study, we have constructed a multiplex panel gathering all the gene abnormalities described by Frampton *et al*.[1]. In addition, to avoid missing mutations by the iPLEX mass spectrometry, we have developed an HRM screening that enables us to detect previously described mutations and confirmed by Sanger sequencing. The pattern of MET mutations found by our team was in the range of those recently published for adenocarcinomas [1, 2]. The 5.3% frequency of exon 14 MET mutations was derived from 150 adenocarcinomas carrying similar mean gene mutation frequencies as those previously reported in France for *KRAS* (23.3%), *EGFR* (9.3%), and *BRAF* (0.3%) [14].

In conclusion, we revealed a 4.9% rate of *MET* exon 14 skipping mutations in 81 European SC patients, which is somewhat lower than that previously published. The high previously reported rate could be explained by the highly selected patient populations studied, *i.e*., the proportion of pleomorphic carcinomas exhibiting an adenocarcinoma component yet with a low rate of other driver mutations like *KRAS*. Furthermore, the 5% rate found in 150 consecutive metastatic adenocarcinomas was within the range of previous reports, thereby validating our detection technique. Even if this SC rate proves the lowest compared to previous published studies, our results emphasize the necessity to perform routine tests in all patients, especially those with SC. However, other driver mutations likely exist and need to be searched for in this cancer subtype.

## MATERIALS AND METHODS

### Patients and tissue tumor collection

For the SC cohort, tissue samples were obtained from surgical lung biopsies of 81 consecutive patients with pulmonary SC who had undergone surgery between 2005 and 2012 in four referral thoracic oncology centers, as previously described [7]. Following central review, only samples exhibiting tumor cellularity >50% were utilized. Each patient signed a consent form as required by national guidelines. The samples were collected according to French legislation along with the ethical codes. A non-opposition for use of tumor sample was obtained from each patient. Clinical data pertaining to demographics and tumor characteristics was extracted from patient medical records. Patients having smoked <100 cigarettes in their lifetime were defined as never smokers, while all others were considered smokers. Tumors were staged following the International Association for the Study of Lung Cancer TNM (Seventh Edition) recommendations [15].

For the adenocarcinoma cohort, all the consecutive FFPE samples with tumor cellularity >50% prospectively screened for mutations as routine procedure (*EGFR*, *KRAS*, *HER2*, *BRAF*, and *PI3KCA)* between June and October 2015, were also screened for MET gene abnormalities.

### Molecular screening for MET gene sequence abnormalities

Overall, 10μm-thick sections were cut from the paraffin blocks. Total DNA was extracted from three sections using the QIAampDNA mini-kit® (Qiagen Inc., Courtaboeuf, France), according to the manufacturer's instructions. Briefly, tissues were disrupted in a lysis buffer. Following paraffin removal, DNA was purified via sequential centrifugation through membrane spin columns. DNA quantity was assessed by measuring the fluorometric absorbanceby means of the Qubit® 3.0 Fluorometer (Thermo Fischer Scientific, Courtaboeuf, France). The quality of the extracted DNAs was confirmed by a HRM cycle threshold below 36 cycles.

We have developed two complementary PCR analysis techniques in order to screen all MET alterations affecting exon 14 splice sites that are indels occurring within splice donor and acceptor sites, and intronic non-coding regions immediately adjacent to exon 14. The first is the MassARRAY iPLEX genotyping technology (Agena Bioscience, San Diego, USA) that enables the synchronous screening of known gene abnormalities in a single run. We have constructed a panel that gathers all the previously reported actionable MET sequence abnormalities resulting in exon 14 alterations^1^. This technique allows detection of single nucleotide mutations (exon 14 MET gene c3082, c3082+1, c3082+2, and c3082+3). This technology proves highly sensitive for facilitating the diagnosis in FFPE samples of limited tissue. The MassARRAY iPLEX procedure involved a three-step process comprising the initial PCR reaction, inactivation of unincorporated nucleotides by shrimp alkaline phosphatase, and a single-base primer extension. The products were subsequently nano-dispensed onto a matrix-loaded silicon chip (SpectroChipII, Agena Bioscience) and finally, the mutations were detected by means of MALDI–TOF (matrix-assisted laser desorption/ionization time-of-flight) mass spectrometry. A data analysis was performed using MassARRAY Typer Analyzer software 4.0.4.20 (Agena Bioscience), which facilitated visualization of data patterns, as well as the raw spectra.

The second technique was a High Resolution Melting (HRM) assay using Lightcycler 480 system (Roche Diagnostics) in order to screen all gene sequence abnormalities of exon 14 of MET gene including indels. HRM was based on two PCRs covering intronic regions before and after exon 14 of the MET gene while using specific primers (PCR 1 forward 5′CATGAGTTCTGGGCACTGGG3′, reverse 5′TAGTTGGGCTTACACTTCGGG3′; PCR 2 forward 5′AGGCTTGTAAGTGCCCGAAG3′, reverse 5′CAATGTCACAACCCACTGAGG3′). If a positive profile was detected, mutations were confirmed using MassARRAY iPLEX technology and Sanger sequencing analysis. For Sanger confirmation, HRM samples were purified with a PCR Purification Kit (Qiagen) and submitted to Cycle Sequencing with BigDye Terminator ready reaction mix (Applied Biosystems Life Technologies), with the same primers used. After purification with a DyeEx 2.0 Spin Kit (Qiagen), samples were analyzed using an ABI Prism 310 Genetic Analyzer (Applied Biosystems Life Technologies).

In order to validate the specificity and sensitivity of the combined HRM and mass spectrometry-based strategy, a subset of 10 tumors (5 with and 5 without MET exon 14 mutations or indels) was also analyzed by next-generation sequencing using the solid tumor solution by Sophia Genetics® based on the xGen Lockdown IDT® probe-based capture technology to enrich whole-genome Illumina-compatible sequencing libraries prepared using the KAPA Hyper Plus Kit. Each patient's whole-genome library contains a unique and different molecular identifier. Targeted resequencing by massively parallel sequencing was performed using an Illumina MiSeq® instrument. Generated raw NGS data was analyzed at the Sophia DDM^TM^ platform (Sophia Genetics, Saint-Sulpice, Switzerland). The five MassArray-identified MET gene sequence abnormalities were retrieved by hybrid capture-based sequencing. Conversely, no other MET abnormalities were identified in the five remaining samples.

### Molecular screening for other gene sequence abnormalities

For the SC cohort, mass spectrometry was used to test 214 mutations affecting 26 oncogenes and tumor suppressor genes (Panel Lungcarta© - MassARRAY iPLEX genotyping technology (Agena Bioscience, San Diego, USA), as previously described [7].

For the adenocarcinomas cohort, routine molecular analysis for *EGFR, KRAS, BRAF, HER2, and PI3KCA* mutations were run using the MassARRAY iPLEX genotyping technology (Agena Bioscience, San Diego, USA), enabling the detection of 159 mutations across the five genes by multiplex PCR single-base extension reactions (iPLEX chemistry). This assay was previously validated using well-characterized FFPE control samples with known mutations (Horizon diagnostics, Amplitech, Compiegne, France) or allele specific PCRs (Lightcycler, Roche). In 2014, the MassARRAY iPLEX genotyping panel involving 159 actionable targets was ISO15189 certified in our center.

### FISH analyses

FISH assays were performed using the MET/CEP7 dual color probe set from Zytovision (Clinisciences, France) according to the manufacturer's instructions. Signals were enumerated in at least 100 tumor nuclei per core. For each core, the mean and standard deviation of copy number per cell of each tested DNA sequence, the percentage of cells with ≥5 copies of MET genes, and the MET/CEP7 ratio were calculated. The 5-copy threshold for positivity has been previously reported to be an independent negative prognostic factor in surgically resected NSCLC [16]. True gene amplification was defined as a MET gene copy number >5 and a MET/CEP7 ratio >2.

## SUPPLEMENTARY TABLE




