# Supplementary material for: MET exon 14 mutations as targets in routine molecular analysis of primary sarcomatoid carcinoma of the lung
Source: Oncotarget. 2017 Mar 21;8(26):42428–37. doi: 10.18632/oncotarget.16403 (PMC5522077; doi:10.18632/oncotarget.16403)
Supplement: Supplementary file 1 [file oncotarget-08-42428-s001.pdf]

MET exon 14 mutations as targets in routine molecular analysis of primary sarcomatoid carcinoma of the lung

SUPPLEMENTARY TABLE

Supplementary Table 1: FISH *MET* results in sarcomatoid carcinoma (n=71).

See Supplementary File 1
