# Supplementary material for: MET exon 14 mutations as targets in routine molecular analysis of primary sarcomatoid carcinoma of the lung
Source: Oncotarget. 2017 Mar 21;8(26):42428–37. doi: 10.18632/oncotarget.16403 (PMC5522077; doi:10.18632/oncotarget.16403)
Supplement: Supplementary file 2 [file oncotarget-08-42428-s002.docx]

**Table S1 : FISH *MET* results in sarcomatoid carcinoma (n=71).**

| Sample | mean *MET* copies per cell | MET/CEP7 ratio | Results |
| --- | --- | --- | --- |
| 1 | 2,3 | 1 | No amplification |
| 2 | 2,1 | 1 | ” |
| 3 | 2,3 | 1,1 | ” |
| 4 | 2,5 | 1 | ” |
| 5 | 6,4 | 1 | Polysomy |
| 6 | 5,1 | 1 | Polysomy |
| 7 | 4 | 1 | No amplification |
| 8 | 3,1 | 1,2 | ” |
| 9 | 2,3 | 1 | ” |
| 10 | 3 | 1 | ” |
| 11 | 3,4 | 1 | ” |
| 12 | 3,2 | 1 | ” |
| 13 | 2,7 | 1 | ” |
| 14 | 2 | 1 | ” |
| 15 | 4,5 | 1 | ” |
| 16 | 2,5 | 1 | ” |
| 17 | 2,3 | 1 | ” |
| 18 | 2,8 | 1 | ” |
| 19 | 2,1 | 1 | ” |
| 20 | 4,1 | 1 | ” |
| 21 | 5 | 1 | Polysomy |
| 22 | 4,2 | 1 | No amplification |
| 23 | 12,4 | 3,1 | **True gene amplification** |
| 24 | 4,5 | 1 | No amplification |
| 25 | 4,9 | 1 | ” |
| 26 | 3,3 | 1 | ” |
| 27 | 3,6 | 1 | ” |
| **28 $** | 2,3 | 1 | ” |
| 29 | 2,7 | 1 | ” |
| 30 | 3,2 | 1,1 | ” |
| 34 | 3,8 | 1 | ” |
| 35 | 2,7 | 1 | ” |
| 36 | 3,7 | 1,1 | ” |
| 37 | 2,6 | 1 | ” |
| 38 | 2,8 | 1 | ” |
| 40 | 13,3 | 6,7 | **True gene amplification** |
| 41 | 3,3 | 1 | No amplification |
| 43 | 15,2 | 5,2 | **True gene amplification** |
| 44 | 3,1 | 1,1 | No amplification |
| 45 | 2,2 | 1 | ” |
| 46 | 3 | 1 | ” |
| 47 | 3,1 | 1 | ” |
| 48 | 5,5 | 2,7 | **True gene amplification** |
| 49 | 3,5 | 1 | No amplification |
| 51 | 2,5 | 1 | ” |
| 52 | 3,1 | 1 | ” |
| 53 | 3 | 1 | ” |
| 54 | 2,6 | 1 | ” |
| 55 | 2,3 | 1 | ” |
| 56 | 2,8 | 1 | ” |
| **57 $** | 2,9 | 1 | ” |
| 58 | 3,5 | 1 | ” |
| 59 | 3,5 | 1 | ” |
| 60 | 3,4 | 1,1 | ” |
| 61 | 3,3 | 1 | ” |
| 62 | 2,4 | 1 | ” |
| 63 | 2,7 | 1 | ” |
| 65 | 2,4 | 1 | ” |
| 66 | 2,1 | 1 | ” |
| 67 | 2,1 | 1 | ” |
| 70 | 6 | 1 | Polysomy |
| **71 $** | 5,2 | 1 | Polysomy |
| 72 | 3 | 1 | No amplification |
| 73 | 2,2 | 1 | ” |
| 74 | 7,5 | 2,3 | **True gene amplification** |
| **76 $** | 5,2 | 1 | Polysomy |
| 77 | 12,1 | 3,3 | **True gene amplification** |
| 78 | 5,1 | 1 | Polysomy |
| 79 | 2,6 | 1 | No amplification |
| 80 | 6,4 | 1 | Polysomy |
| 81 | 4,8 | 1,7 | No amplification |

Sample $ : Sample with Met Exon 14 mutation

CEP7 : centromere 7
